# A Comprehensive Exploration on Occurrence, Distribution and Risk Assessment of Potentially Toxic Elements in the Multi-Media Environment from Zhengzhou, China

**DOI:** 10.3390/toxics11020140

**Published:** 2023-01-31

**Authors:** Jialu Li, Qiting Zuo, Hao Hu, Feng Feng, Hongtao Jia, Yingxin Ji

**Affiliations:** 1 Yellow River Conservancy Technical Institute, Kaifeng 475000, China; 2 Henan Engineering Research Center of Project Operation and Ecological Security for Inter-Basin Regional Water Diversion Project, Kaifeng 475004, China; 3 Henan Engineering Technology Center for Water Resources Conservation and Utilization in the Middle and Lower Reaches of Yellow River, Kaifeng 475004, China; 4 School of Water Conservancy Engineering, Zhengzhou University, Zhengzhou 450001, China; 5 Zhengzhou Key Laboratory of Water Resource and Water Environment, Zhengzhou 450001, China; 6 Henan International Joint Laboratory of Water Cycle Simulation and Environmental Protection, Zhengzhou 450001, China

**Keywords:** potentially toxic elements, road dust, roadside soils, sediments, ecological risks

## Abstract

Road dust (RD), roadside soils (RS) and river surface sediments (sediments) are important materials for evaluating contaminant levels in urban areas. This study aims to investigate the contaminant characteristics, pollution levels and ecological risks of RD, RS and sediments of potentially toxic elements (PTEs), including Cr, Ni, Cu, Zn, As, Cd, Hg and Pb, in the central urban area of Zhengzhou. Results reveal that RD shows a higher concentration of PTEs when compared to the other two environments. The spatial distribution characteristics suggest that PTEs in RD, RS and sediments may come from different sources. The geo-accumulation index (*I_geo_*) was used to describe that the RD was moderately to extremely contaminated with Cd and Hg, while both RS and sediments were significantly enriched with Cd and Hg. For RD, RS and sediments, the potential ecological risk (RI) demonstrates a high potential ecological risk from Cd and Hg. Overall, PTEs in Zhengzhou road dust present a moderate risk.

## 1. Introduction

With the development in social economies, cities attract large scale population because of their aggregation of social resources and a good living environment. The world’s total population increased from 2.53 billion to 7.44 billion from 1950 to 2018, and the proportion of urban population increased from 30% to 54%. The world’s population is expected to reach 9.55 billion by 2050, and the number of urban people will increase by 2.5 billion, accounting for 66% of the total population. Human society has gradually shifted from a society dominated by agriculture to a modern society dominated by industry, commerce and service industries with large populations gathering in cities. However, economic development, population growth, urban expansion and social transformation have greatly changed the natural environment in which we live. Meanwhile, human activities such as production and consumption during the development of society constantly increase the pollution load. Among the pollutants, potentially toxic elements (PTEs) have attracted much attention because of their persistence and resistance to degradation [1,2]. Road dust (RD), roadside soils (RS) and river surface sediments (sediments) are important components of the urban ecological environment. They are closely related to each other, and all of them are “source” and “sink” of PTEs in the urban environment.

Road dust (RD) plays an important role in urban material circulation, and its quality has a significant impact on the quality of the river, soil and ecological environment and human health. Previous studies show that RD not only receives and purifies various pollutants, but also is the secondary pollution source of urban water, air, soil and other environmental medium [3,4,5]. Under the action of external forces, RD will re-suspend into the air and endanger human health through breathing and skin contact. As a result of wind or man-made transport, RD deposits on the surface of soil and pollutes the soil and plants through infiltration. Pollutants accumulated in RD can be re-released into the surface or underground water through rainfall, surface runoff or under suitable environmental conditions, and eventually harm human health through the food chain. In general, the contents of PTEs in urban RD are much higher than in rural or agricultural soil [6,7].

Roadside soils (RS) are composed of minerals, organic matter (humus), biomass, air and water. Similar to RD, the contents of PTEs in the RS are affected by various human activities (e.g., vehicle emissions, industrial emissions and domestic refuse incineration). RS is not only the main source but also the storage of RD. Both RD and RS can be used to monitor the pollution degree of PTEs in urban areas [8,9,10]. The health of sanitation workers, residents living near roads and pedestrians will be affected by long-term exposure to RS containing PTEs through breathing, ingestion and skin contact. Therefore, it is of great significance to study the PTE contents in RS to evaluate the pollution degree of urban PTEs.

Sediments, as an important ecological component of water bodies, play a key role in maintaining the nutritional status, provide a place for biochemical cycles and also are the foundation of the food chain. Hence, sediments are important sites for many chemical and biological processes in water and are indispensable components of ecological integrity. Sediments are both the source and sink of pollutants in water, which is an important indicator reflecting the water environment. After entering the water, most of the PTEs are adsorbed by sediments through complexing with the organic matter, clay minerals and sulfide. Studies have found that the contents of PTEs in sediments were 638–10,349 times higher than those in the overlying water of Taihu [11]. PTEs cannot be decomposed naturally in water, which are different from organic pollutants. When environmental factors (e.g., wind and wave disturbances, pH, ORP, DO and microbial activity) change, PTEs in the sediments are prone to re-suspend and then re-enter into the overlying water, resulting in secondary pollution [12,13]. Urbanization and industrialization have increased the number of PTEs discharged into urban rivers.

The pollution characteristics of PTEs in a single environmental medium have been studied extensively. However, there are few comprehensive studies and comparative analyses on the three environmental media. Zhengzhou city, the capital of Henan Province, is one of the mega cities in China, and an important core city in central China, as it is a major comprehensive national transportation hub. Zhengzhou is also one of the most densely populated cities in China. In 2017, the population density of the central urban area of Zhengzhou reached 3665.25 people/km^2^. The intensity of human activities is great. Zhengzhou lies across the Yellow River and Huai River basin, and the river system is well developed. Previous studies on Zhengzhou have mostly focused on the river water pollution, and there are few studies on road dust and roadside soils, especially on the occurrence of PTEs in Zhengzhou. The main aims of this study are to analyze the pollution characteristics of PTEs in road dust, roadside soils and river surface sediments comprehensively, and to compare the ecological risks between different environmental medium based on the following aspects: (1) contents and spatial distribution of PTEs (Cr, Ni, Cu, Zn, As, Cd, Hg and Pb) in RD, RS and sediments, (2) comparison of the PTE contents in RD, RS and sediments and (3) assess the ecological risks of RD, RS and sediments based on *I_geo_* and RI.

## 2. Materials and Methods

### 2.1. Study Area

**Figure 1:**
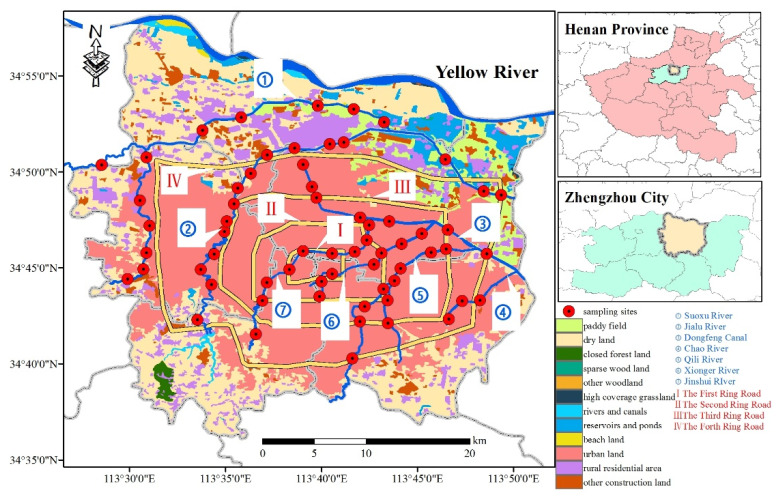
Location of the study area.

Zhengzhou city (112°42′–114°14′ E, 34°16′–34°58′ N) is located in the lower reaches of the Yellow River, north of central Henan Province. The study area is the central urban area of Zhengzhou, including Zhongyuan District, Erqi District, Guanchenghuizu District, Jinshui District and Huiji District, with a total area of 1018.58 km^2^. The geographical location of Zhengzhou city is shown in Figure 1. According to the land use of Zhengzhou in 2018 (Figure 1), the areas of paddy field, dry land, closed forest land, sparse woodland, high coverage grassland, rivers and canals, reservoirs and ponds, beach land, urban land, rural residential area and other construction land are 53.62, 280.75, 3.95, 0.20, 0.10, 2.84, 15.07, 36.97, 7.34, 487.49, 100.87 and 38.49 km^2^, respectively. In the urban area of Zhengzhou, there are mainly seven rivers, which are Jialu River, Dongfeng Canal, Qili River, Suoxu River, Jinshui River, Xionger River and Chao River.

### 2.2. Sampling and Sample Preparation

The spatial distribution characteristics of pollutants in RD, RS and sediments will show heterogeneity under the influence of different types and intensities of human activities. Therefore, according to the basic conditions of Zhengzhou central urban area, such as physical geography, river distribution, traffic conditions and urban development, combined with the grid distribution point method and function area distribution method, 65 sampling points were set up at the intersection of rivers and roads (Figure 1). The RD, RS and sediments samples were collected in October 2019. Since road dust and roadside soils are susceptible to rainfall and wind, sampling should be carried out in at least one week of clear weather to ensure representation of the samples. The collection processes of each environmental media are as follows:

RD: 5–6 sub-samples from both sides of the road were collected randomly using a combination of a clean plastic brush, a plastic dustpan and a vacuum cleaner (Philips, FC8471, self-contained filter). After removing impurities such as bricks, stones, fallen leaves and other debris, they were mixed evenly and stored in a light-proof self-sealing bag, then recorded as a sampling point.

RS: At each sampling site, topsoils with a depth of 0–3 cm were collected using a shovel within a range of 2 m from the edge of the road, and a total of four sub-samples were collected within 1 m of each other. After removal of gravel, animal and plant residues and other debris, the four sub-samples were mixed and stored in a light-proof self-sealing bag. Additionally, at least 250 g of samples was collected at each sampling site.

Sediments: There are no sediments in hard bed rivers; therefore, it is impossible to collect the sediments sample in these rivers. A total of 46 river surface sediments were collected. At each site, surface sediment samples with a depth of 0–5 cm were collected using a Petersen dredge and four sub-samples (5 m apart) were collected and mixed on site, and then also stored in a light-proof self-sealing bag.

All the collected samples (65 RD samples + 65 RS samples + 46 sediments samples) were sent to the laboratory, vacuum freeze-dried at −40 °C, ground through a 100 mesh sieve and stored in 4°C until analysis. The collection and pretreatment of all the samples were in accordance with the standards issued by MEE of China (the technical specification for soil environmental monitoring (HJ/T 166-2004) and water quality guidance on sampling techniques (HJ 494-2009)).

### 2.3. Sample Analysis

In this study, eight kinds of PTEs with high ecological and health risks were selected for analysis and determination; namely, Cr, Ni, Cu, Zn, As, Cd, Hg and Pb. The total contents of PTEs were determined according to the EPA 3052 method. A mixture of acids (6 mL HNO_3_ + 3 mL HF + 1 mL H_2_O_2_) was used for microwave digestion (MARS CEM, Matthews, NC, USA), and then determined by ICP-MS (iCAP Q, Thermo Scientific, Waltham, MA, USA).

### 2.4. Quality Assurance and Quality Control (QA/QC)

The containers used in this study were soaked in 20% HNO_3_ solution (*v*/*v*) for 24 h before use, and then rinsed with ultra-pure water (Milli-Q, *ρ* > 18 MΩ·cm).

In order to ensure the accuracy and precision of the results, a standard sample was used to monitor the results among every 20 samples during the measurement. All the PTEs were measured three times, and the results were expressed as the mean values of three parallel measurements (standard deviation < 5%).

The North China Plain soil standard (GBW07427 GSS-13) was used as the quality control sample. The recoveries of all the PTEs in this study were between 89 and 110%.

Blank samples were measured to eliminate the influence of background values.

### 2.5. Assessment Methods for PTEs

#### 2.5.1. *I_geo_*

The index of geo-accumulation (*I_geo_*) is a method proposed by Müller [14] that can quantitatively evaluate the pollution degree of PTEs in various environmental media, and this method takes into account the effect of diagenesis on background values and human activities on the PTEs contents [15]. The equation for *I_geo_* is as follows:

(1) $$I_{geo}=log_{2}\frac{C_{i}}{1.5\times B_{i}}$$

where *C_i_* represents the measured content of metal *I* and *B_i_* represents the background value of metal *i*. In order to minimize the variation in environmental background values caused by petrogenesis, the constant “1.5” is introduced. The seven-level classification of *I_geo_* is defined and shown in Table S2.

#### 2.5.2. RI

The potential ecological risk index (RI) is widely used to evaluate the pollution level and ecological risk of PTEs in various environmental media [16]. The calculation equation for RI is presented as follows:

(2) $$\mathrm{RI}=\sum E_{r}^{i}=\sum T_{r}^{i}\times C_{f}^{i}=\sum T_{r}^{i}\times C_{i}/B_{i}$$

where $C_{f}^{i}$ is the pollution index of metal i, the meanings of C_i_ and B_i_ are consistent with those defined above, $E_{r}^{i}$ is the potential ecological risk index of metal i and $T_{r}^{i}$ is the toxicity response factor of metal i. In this study, the $T_{r}^{i}$ for Cr, Ni, Cu, Zn, As, Cd, Hg and Pb are 2, 5, 5, 1, 10, 30, 40 and 5, respectively. The grades of RI are shown in Table S3.

Excel2016, Origin 2021b, Suffer11, arcGIS and SPSS22 software were used for statistical tests, plotting and analysis. All spatial distributions were interpolated by the inverse distance (IDW) method.

## 3. Results and Discussion

### 3.1. Contents of Potentially Toxic Elements

#### 3.1.1. Road Dust

Descriptive statistics of PTE concentrations in Zhengzhou road dust are presented in Table 1. Among them, the skewness values of Ni, Cu, Cd and Hg were greater than three, indicating that these elements have high contamination points. Except for As, the median of the other elements was lower than the mean, revealing that there were outliers for these metals. According to the coefficient of variation (CV), PTEs can be classified into four categories: CV < 0.2, which indicates low variability; 0.2 ≤ CV < 0.5, which is regarded as moderate variability; 0.5 ≤ CV < 1, which indicates high variability; and CV ≥ 1, which is considered very high variability. In this study, concentrations of Hg showed maximum variability with a CV of 1.07; the CVs of Ni, Cu and Cd showed high variability; and the CVs of Cr, Zn, As and Pb showed moderate variability.

Due to the lack of relevant research and data on the PTE background values of road dust, roadside soils and river sediments in Zhengzhou, the background value of Henan Province soil was used as the reference value to compare and evaluate the PTE contamination in the samples [17]. The mean concentrations of PTEs exceeded the corresponding background values. In particular, the mean concentrations of Cd and Hg were 8.24 and 4.12 times higher than the corresponding background values, respectively. This indicated that the above-mentioned PTEs may come from anthropogenic sources.

**Figure 2:**
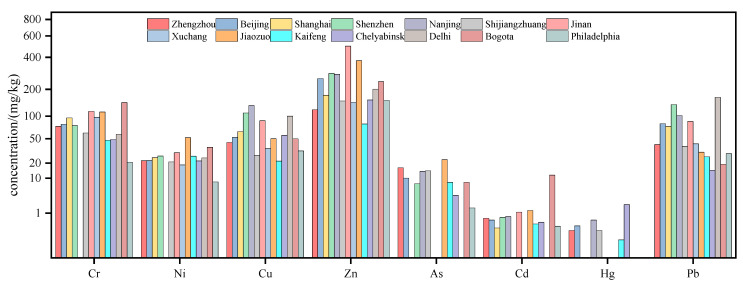
Comparison of PTEs (mg/kg) in road dust with the previous literature. (Beijing [18], Shanghai [19], Shenzhen [20], Nanjing [21], Shijiangzhuang [22], Jinan [23], Xuchang [24], Jiaozuo [25], Kaifeng [26], Chelyabinsk [27], Delhi [28], Bogota [29] and Philadelphia [30].)

Comparing the PTE concentrations in road dust of Zhengzhou with different cities in China and other countries (Figure 2), it was found that the mean concentrations of PTEs in Zhengzhou road dust were generally lower than other mega cities in China, such as Beijing, Shanghai and Shenzhen. The mean concentration of As was higher than that in Beijing and Shenzhen, which may be due to the higher degree of urbanization and lower consumption of arsenic-containing pesticides in Beijing and Shenzhen. Both Shijiazhuang and Jinan are capital cities in northern China, and their socio-economic and meteorological conditions are similar to those of Zhengzhou. Except for Co, the mean concentrations of other elements were close to those in Zhengzhou. Compared with other cities in Henan Province, the mean concentrations of PTEs in Zhengzhou road dust were alike to those in Xuchang City. Jiaozuo City is rich in mineral resources. There are a large number of heavy industrial enterprises in the city such as mineral smelting and processing. Except for Pb, the mean concentrations of PTEs in Zhengzhou road dust were lower than those in Jiaozuo. In Kaifeng City, which is adjacent to Zhengzhou, the mean concentration of Ni was slightly higher than that in Zhengzhou, while the mean concentrations of other metals were far lower. Chelyabinsk is the largest heavily industrial city in the Ural region of Russia. The concentrations of Cr, As and Pb in Chelyabinsk road dust were lower than those in Zhengzhou, while the concentration of Hg was much higher. Delhi, the capital of India, is one of the most populous cities in the world. The concentrations of Zn, Cu and Pb in Delhi road dust were much higher than those in Zhengzhou. As the capital of Colombia, Bogota is the industrial center of Latin America. However, its physical geography and climatic conditions are different from those of Zhengzhou, and the mean concentrations of PTEs in Bogota road dust were generally lower than those in Zhengzhou. The above results showed that the concentrations of PTEs in Zhengzhou road dust were at a medium level compared with other cities.

**Figure 3:**
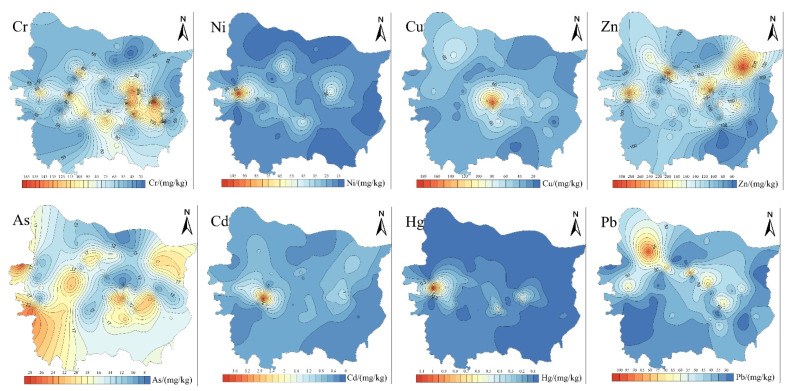
Spatial distribution of PTE concentration in road dust of Zhengzhou.

Figure 3 illustrates the spatial distribution characteristics of PTEs in Zhengzhou road dust. The concentrations of Cr, Ni, Hg and Pb were relatively high at the same sampling site, which was because construction was taking place near the sampling site. The extensive use and disposal of ceramics, paint, cement, steel and other engineering materials during the construction increased the concentrations of Cr, Ni and Hg in the road dust. Moreover, the exhaust emissions from vehicles and machinery at the construction site also increased the concentrations of Cr and Pb. The spatial distribution characteristics of As showed that the concentrations in the southwest were higher. The high value area used to be farmland, but now it is basically bare land. The application of arsenic-containing pesticides, herbicides, defoliants and hybrid rice malecides increased the concentrations of As in soils [31], which then gradually weathered and became part of road dust. Near the sampling site with the highest Cd value, Jialu River was undergoing environmental dredging and highly polluted sediments accumulated on the river bank, increasing the Cd concentration. Moreover, there is a bus station under the overpass; traffic flow, frequent braking and tire wear have increased the Cd concentration in road dust. The spatial distribution characteristics of Pb were related to traffic conditions. Even though the use of leaded gasoline has been completely banned in China since 2005, lead in the vehicle emissions still increases the Pb concentrations in the RD. Vehicle exhaust emissions are an important source of Pb in road dust.

#### 3.1.2. Roadside Soils

Descriptive statistics about PTE concentrations in the Zhengzhou RS samples are summarized in Table 2. The CVs of Zn, Cd and Hg were greater than 0.3, indicating that these PTEs may come from different sources. The mean concentrations of PTEs were higher than the corresponding soil background values in Henan Province, especially Cd, whose mean concentration was 5.5 times higher than that of the corresponding background value. Significant enrichment in Cd indicated that Cd may mainly come from anthropogenic sources. Although the mean concentrations of all the investigated PTEs in the RS were lower than the Level II of China’s soil environmental quality standard, the Cd concentration in 12.3% of the sampling sites was higher than the corresponding Level II standard value, and 7.0% of the sampling sites‘ Cd concentration was higher than the corresponding Level III standard value. It is suggested that Cd may have adverse effects on the growth of roadside plants and human health when exposed to the RS for a long time.

Compared with the published literature on other cities at home and abroad (Table 3), the mean concentrations of Cr, Ni and Zn were similar to those in Beijing, while the mean concentration of Pb was lower, which may reflect the impact of traffic conditions on Pb concentration in RS. The mean concentration of As was higher, which may be related to agricultural activities. Compared with other megacities in China (e.g., Beijing and Shanghai), agricultural activities around Zhengzhou are still active. Xi’an and Zhengzhou are similar in population size, traffic conditions and economic and social development, and the PTE concentrations in the RS of the two cities were also similar. Compared with developed countries (e.g., Australia and Canada), PTE concentrations in the RS in developing countries (e.g., China and India) were higher. However, the concentrations of Ni, Cu and Pb in the RS of Siena were relatively high, which may be due to the long road service life (at least 35 years) and the large traffic flow (about 50,000 vehicles/day). In addition, the mean concentration of Cd in this study was significantly higher than that in other cities, which may be related to more construction activities in Zhengzhou.

**Figure 4:**
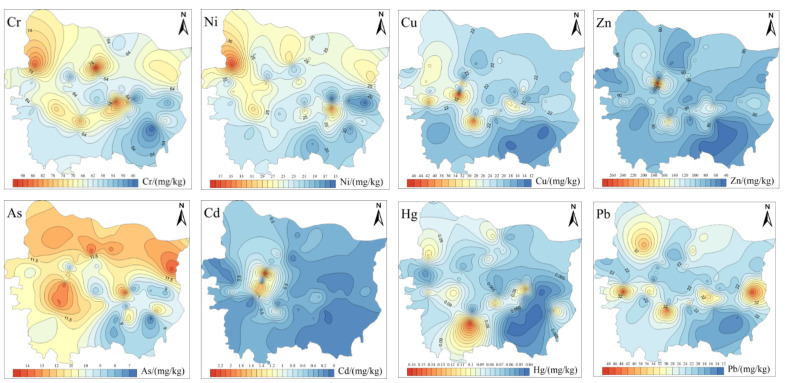
Spatial distribution of PTE concentrations in roadside soils of Zhengzhou.

The spatial distribution characteristics of PTEs in the RS may be related to wind speed, wind direction, road width and distribution and height of buildings in the area. Figure 3 illustrates the spatial distribution characteristics of PTEs in RS of Zhengzhou. As shown in Figure 4, the concentrations of most PTEs were generally higher in the west than in the east, which may be because of the wind direction (the dominant wind direction of Zhengzhou in autumn is a southwest wind), and wind direction is an important factor affecting the concentrations of PTEs in the RS. The spatial distribution characteristics of Cr and Ni in the RS of Zhengzhou were similar, while the spatial distribution characteristics of Cu, Zn and Cd in the RS were similar. The anthropogenic sources of Cu mainly include the wear of automobile engines and parts (such as thrust bearings, bushings and metal bearings). Zn in the soils mainly comes from automobile batteries, carburetor, lubricating oil and automobile tire wear. The Cd concentration in the RS is related to the pH and ORP of the soils. In alkaline medium (pH > 7) and an oxidized environment, Cd can form stable minerals, such as Otavite (CdCO_3_), montmorillonite (CdO) and cadmium hydroxide (Cd(OH)_2_) [38,39]. Due to the lack of large-scale industrial activities near the hotspot, the high Cd concentration nearby may be because of the use of lubricants, in addition, on rough roads, tire wear increases the Cd concentration in the RS. There is a large bus station near the hotspot, so the concentrations of Cu, Zn and Cd in the RS were mainly related to the traffic conditions. The spatial distribution characteristics of As in the RS of Zhengzhou showed a trend of being higher in the north than in the south, and higher in the west than in the east. This may be because that compared with the southeastern part, there is more green space, urban parks and farmland in these areas. The use of arsenic pesticides (such as calcium arsenate and sodium arsenite) and phosphate fertilizers increased the As concentration in the RS. The spatial distribution characteristics of Hg in the RS were different from that of RD. Previous studies [40,41] have shown that inorganic mercury and methylmercury were widespread in the sludge of sewage treatment plants, and the Hg concentrations in the sludge were relatively high. There is a sewage treatment plant near the Hg hotspot, and the accumulation of sludge may have increased the Hg concentration in the RS. The highest concentration of Pb in the RS was 46.96 mg/kg, which was 2.4 times higher than the corresponding background value. The Pb hotpots in the RS were related to industrial and vehicle emissions. Furthermore, the number of new energy vehicles in Zhengzhou reached 85,000 in 2020, and the Pb in the batteries also intensified the Pb pollution in the RS. Hence, vehicle emissions may be one of the main factors for the accumulation of PTEs in the RS in Zhengzhou. In general, the spatial distribution characteristics of PTEs in the RS of Zhengzhou were related to many factors, such as road density, location of the main road, industrial type and topography and geomorphology of the study area.

#### 3.1.3. River Surface Sediment

Table 4 shows the descriptive statistics of PTE concentrations in the surface sediments of the rivers in Zhengzhou. Except for As, the mean concentrations of other PTEs were higher than their corresponding background values, especially Cd and Hg, which were 3.5 and 2.6 times higher than the corresponding background values, respectively.

The sediment quality guidelines (SQGs) were developed based on benthic toxicity tests to assess the adverse biological effects of contaminants in sediments and to determine tolerable concentrations of contaminants in sediments, which were proposed by MacDonald et al. [42]. In order to determine whether PTEs in sediments pose a threat to aquatic ecosystems, the SQGs propose two sets of criteria, including threshold effect level (TEL), probable effect level (PEL), effects range low (ERL) and effects range median (ERM). When the pollutant concentration is lower than the ERL value, there is no adverse effect on organisms, while ERM refers to the threshold at which adverse biological effects may occur. Compared with the corresponding thresholds of SQGs, the mean concentrations of PTEs were lower than the corresponding PEL and ERM values, indicating the surface sediments of rivers in Zhengzhou may not cause acute toxicity to benthic organisms. However, the mean concentration of Ni exceeded the corresponding TEL and ERL values, at 1.12 and 1.24 times higher than the TEL and ERL values, respectively. The mean concentration of Cr was between the corresponding TEL and ERL value. Therefore, Ni and Cr in the sediments may have adverse biological effects and chronic toxicity. The mean concentration of As was higher than the corresponding ERL value, but did not exceed the TEL value, and the risk was low.

The results of comparing the mean concentrations of PTEs in Zhengzhou river surface sediments with other rivers within and outside the country are shown in Table 5. At the tributaries of Shaying River, the mean concentrations of PTEs in the river surface sediments of Zhengzhou were similar to that of Shaying River. However, after a series of river ecological restoration projects, the PTE concentrations in river surface sediments of Zhengzhou decreased significantly, especially those of Cu, Zn, Cd and Pb, whose mean concentrations were much lower than that of Kaifeng, which is adjacent to Zhengzhou. The water quality of urban rivers in Shanghai and Wuhan was more strongly affected by human activities, and the PTE concentrations in these river sediments were higher. The water quality of urban rivers in Qingdao was mainly affected by Jiaozhuo Bay, and the PTE concentrations in the sediments were lower than those of other industrial/urban ports and estuaries. The PTE concentrations in river surface sediments of Zhengzhou were close to those of Qingdao, which could indicate that the ecological restoration projects in Zhengzhou have made some progress to a certain extent. However, compared with the rivers in developed countries, the PTE pollution in river surface sediments in Zhengzhou was still serious. Tsurumi River has undergone a comprehensive improvement since the 1970s. However, it is still affected by urbanization and industrialization, and the PTE concentrations in the sediments were relatively high.

**Figure 5:**
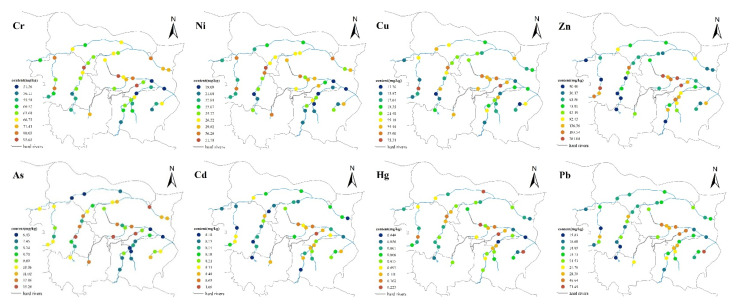
Spatial distribution of PTE concentration in river surface sediments of Zhengzhou.

Figure 5 illustrates the spatial distribution characteristics of PTEs in river surface sediments of Zhengzhou. In this study, the mean concentration of Cr in the rivers of Zhengzhou decreased in the order Xionger River > Jinshui River > Dongfeng Canal > Jialu River > Suoxu River > Chao River > Qili River. The Cr concentrations in all sampling sites exceeded the corresponding TEL value, while the Cr concentration in 6.5% of sampling sites was higher than the ERL value. Both Cu and Zn are essential micronutrients for aquatic organisms in natural water and sediments. Although they have low concentrations of micronutrients in natural water, they can become toxic to aquatic organisms when their concentrations exceed the corresponding threshold. As shown in Figure 4, the spatial distribution characteristics of Cu and Zn in the sediments of Dongfeng Canal, Jinshui River, Xionger River, Qili River and Chao River were similar, while the enrichment degrees of Cu in the sediments of Suoxu River and Jialu River were higher. The mean concentration of Cu in the sediments decreased in the order Xionger River > Jinshui River > Dongfeng Canal > Suoxu River > Jialu River > Qili River > Chao River. The concentration of Cu in 13.0% of sampling sites was higher than the corresponding TEL value. In contrast, the mean concentrations of Zn in the sediments decreased in the order of Xionger River > Dongfeng Canal > Jinshui River > Suoxu River > Qili River > Chao River > Jialu River, and the Zn concentration in 28.3% of sampling sites was higher than the corresponding TEL value, while the concentration in 17.4% of sampling sites was higher than the corresponding ERL value, especially in Xionger River, where Zn in the sediments may have adverse effects on benethic organisms. As and Cd have been confirmed by the US EPA as carcinogenic to humans and also have potential risks to ecological communities. Research by Sadip has showed that [51], compared with other PTEs, Pb at low concentrations still posed a threat to aquatic organisms. The spatial distribution characteristics of Cd and Pb were similar; that is, the concentrations of Cd and Pb in Xionger River, Dongfeng Canal and Jinshui River were higher, and in Suoxu River and Jialu River, they were lower. Meanwhile, the concentrations of Cd and Pb in the sampling site XE-2 exceeded the corresponding TEL value. Hg is a kind of highly toxic pollutant with a low concentration in natural water, generally less than 0.1 μg/L. The spatial distribution of Hg decreased in the order Xionger River > Chao River > Dongfeng Canal > Suoxu River > Jinshui River > Jialu River > Qili River.

### 3.2. Comparison of PTE between Road Dust, Roadside Soils and Surface Sediments

**Figure 6:**
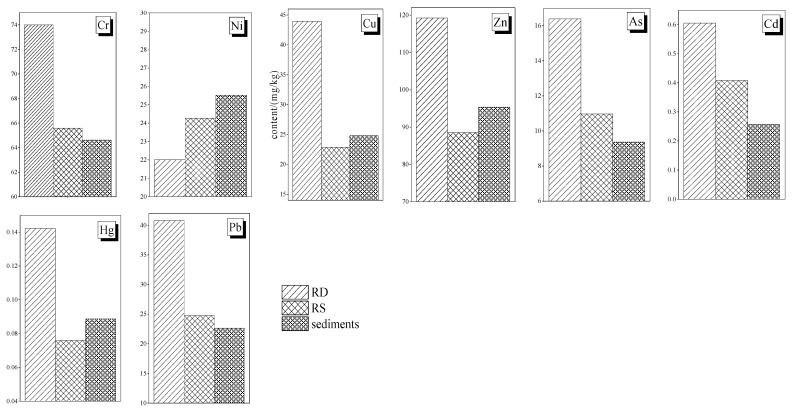
Mean concentration of different PTEs in road dust, roadside soil and surface sediment samples.

The mean concentrations of PTEs in RD, RS and sediments are compared in Figure 6. Except for Ni, the mean concentrations of other PTEs in the RD were the highest, which was consistent with the results of previous studies [7,52,53]. The mean concentration of PTEs in RD was 1.72 and 1.88 times higher than that in RS and river surface sediments, respectively. Compared with RS and river surface sediments, RD is affected by many types of human activities directly, and has a smaller particle size and larger specific surface area, which make it easier to absorb PTEs from the environment. In addition, the content of microorganisms in RD is low, which makes the degradation rate of PTEs slower and the enrichment degree in RD higher. The mean concentrations of Cr, As, Cd and Pb in river surface sediments were lower than those in RD and RS. The water depth of urban rivers is generally shallow, and material exchange between overlying water and sediments is frequent. The PTEs in the sediments will re-enter the overlying water through resuspension, diffusion and desorption, which will reduce the PTE concentrations in the sediments. Furthermore, since the samples were collected in autumn, the decomposition of plant residues in the rivers further drives the release of PTEs from the sediments into the overlying water. In order to eliminate urban black odor water, Zhengzhou government has launched a series of comprehensive treatment projects. After environmental protection dredging, a large number of PTEs and other pollutants accumulated in the sediments were removed, which cleared the contaminated sediments effectively and reduced the pollutant concentrations and ecological risks in river sediments, especially in urban river sediments.

### 3.3. Pollution Assessment

#### 3.3.1. *I_geo_*

**Figure 7:**
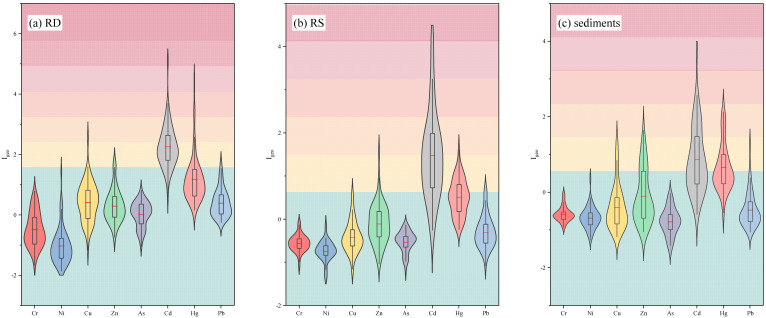
Violin plots of geo-accumulation index (*I_geo_*) for PTEs in road dust (**a**), roadside soils (**b**) and surface sediments (**c**) of Zhengzhou. The middle red lines represent the mean value. The ends of the box represent the 25th and 75th quantiles.

The *I_geo_* values calculated for each PTE in road dust, roadside soils and river surface sediments of Zhengzhou are presented in Figure 7. The mean *I_geo_* values of PTEs in road dust were ranked as follows: Cd > Hg > Cu > Pb > Zn > As > Cr > Ni. The contamination level of Cd was the highest among the PTEs in this study, followed by Hg and Cu. According to the standard classification by *I_geo_*, the mean *I_geo_* values of the road dust indicated that they were moderately to heavily contaminated by Cd (2.25), moderately contaminated by Hg (1.18), uncontaminated to moderately contaminated by Cu (0.42), Pb (0.39), Zn (0.30) and As (0.02) and uncontaminated by the other PTEs. Previous studies have also shown a heavier contamination of Cu, Zn, Pb and Cd in road dust of other Chinese cities. Figure 7 also shows that the *I_geo_* values of PTEs varied widely at each sampling site. The maximum *I_geo_* values of Cd, Hg, Zn, Cu, Pb and Ni were 5.09, 4.43, 3.41, 2.57, 1.73 and 1.44, respectively, which indicated that the PTE pollution load in these sampling sites was relatively heavy.

The mean *I_geo_* values of PTEs in roadside soils decreased in the order: Cd > Hg > Zn > Pb > Cu > As > Cr Ni. The mean *I_geo_* values of roadside soils indicated a moderate contamination of the roadside soils by Cd (1.48), it indicated that they were uncontaminated to moderately contaminated by Hg (0.51) and the *I_geo_* values of other PTEs were negative. Additionally, according to the mean *I_geo_* values of PTEs in river surface sediments, the *I_geo_* grade for Cd and Hg was uncontaminated to moderately contaminated. The *I_geo_* values for other PTEs exhibited a mean value < 0, which showed that no contamination was found in the case of these PTEs. Cd was found at the highest contamination level in road dust, roadside soils and river surface sediments.

#### 3.3.2. RI

**Figure 8:**
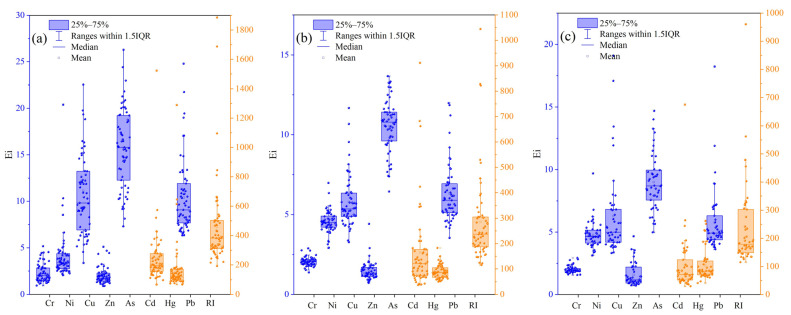
Box plots of $E_{r}^{i}$ and RI in RD (**a**), RS (**b**) and sediment (**c**) samples from Zhengzhou.

A comparison of the mean values of $E_{r}^{i}$ of road dust, roadside soils and surface sediments is presented in Figure 8. As shown in Figure 8a, the $E_{r}^{i}$ values of Cd in road dust were in the range of 67.65–1523.23, with a mean value of 245.54, indicating a high risk, while the mean $E_{r}^{i}$ value of Hg was 167.31, corresponding to high risk. The $E_{r}^{i}$s for Cr, As and Pb in all sampled road dust were below 40, indicating low risk. Additionally, the mean $E_{r}^{i}$ value of Cd in roadside soils was 165.41, indicating high risk, and the $E_{r}^{i}$ values in almost 32% of sampled roadside soils exceeded 160, corresponding to very high risk. The mean $E_{r}^{i}$ value of Hg was 89.24, indicating considerable risk. Regarding Cd and Hg, in almost all the sampled surface sediments, the $E_{r}^{i}$ value exceeded 80, and the mean $E_{r}^{i}$ values corresponded to considerable risk. Figure 8 also illustrates the RI values in road dust, roadside soils and surface sediments. The mean RIs for road dust, roadside soils and surface sediments were 542.38, 331.94 and 281.79, respectively. These values indicated that road dust was classified as considerable risk, and roadside soils and surface sediments showed moderate risk. As shown in Figure 8b, the roadside soils in most sampling sites were at moderate risk, while in the southeast, they were at low risk. Figure 8c illustrates that 58.70% and 32.61% of the sampled surface sediments showed low and moderate risks, respectively. Obviously, due to the high accumulation and the strong toxicity of Cd and Hg, these two elements had the highest contribution rates to RI, and played a major role in increasing the ecological risk in the urban environment.

## 4. Conclusions

In this study, the pollution characteristics and ecological risks of PTEs in road dust, roadside soil and river surface sediment samples from central urban areas of Zhengzhou were determined. The mean concentrations of Cr, Cu, Zn, Cd, Hg and Pb in road dust, roadside soil and river surface sediment samples were all higher than the corresponding background values. The spatial distribution characteristics of PTE concentrations indicated that the main anthropogenic sources of PTEs in Zhengzhou were industrial production, agricultural activities, vehicle exhaust emissions and engineering construction. In general, the mean concentrations of PTEs were the highest in road dust samples. In this case, Cd and Hg, which were derived from multiple anthropogenic sources, were the main polluting elements in road dust, as their mean *I_geo_* values ranged from the classes of moderately to heavily contaminated. The *I_geo_* values of Cd and Hg in roadside soils and surface sediments ranged from the classes of uncontaminated to moderately contaminated. Regarding the potential ecological risk assessment, in road dust samples, Cd and Hg posed a high risk. The mean $E_{r}^{i}$ values in roadside soils and surface sediments indicated that Cd and Hg caused moderate or considerable risk at many sites. Therefore, for the health of the urban ecosystem, more attention should be paid to the levels of Cd and Hg. According to RI values, the ecological risk from road dust was comparatively higher.

## Figures and Tables

**Table 1:** PTE concentrations (mg/kg) in road dust collected from Zhengzhou.

	Cr	Ni	Cu	Zn	As	Cd	Hg	Pb
Max	165.22	108.91	174.88	307.21	27.35	3.76	1.10	97.21
Min	30.29	11.09	13.28	54.30	7.61	0.17	0.057	24.89
Mean	74.00	22.01	43.93	119.25	16.39	0.61	0.14	40.78
Median	64.64	18.18	39.65	104.52	16.41	0.49	0.10	36.16
SD ^1^	30.98	14.27	23.15	52.30	4.35	0.47	0.15	15.00
Skewness	1.08	4.14	3.05	1.90	0.058	5.07	4.88	1.73
Kurtosis	0.51	22.02	15.45	3.93	−0.41	32.69	27.75	3.31
CV ^2^	0.42	0.65	0.53	0.44	0.27	0.77	1.07	0.37
BV ^3^	63.80	26.70	19.70	60.10	10.40	0.074	0.034	19.60

^1^ standard deviation, ^2^ coefficient of variance, ^3^ background value: CNEMC 1990.

**Table 2:** Descriptive statistics for PTE concentrations (mg/kg) in roadside soils samples of Zhengzhou.

	Cr	Ni	Cu	Zn	As	Cd	Hg	Pb
Max	91.96	37.29	45.98	265.64	14.22	2.25	0.16	46.96
Min	44.03	15.53	12.89	44.15	6.70	0.09	0.04	13.88
Mean	65.58	24.27	22.88	88.57	10.97	0.41	0.08	24.76
SD	8.93	3.86	6.43	34.77	1.75	0.40	0.02	7.17
CV	0.14	0.16	0.28	0.39	0.16	0.99	0.30	0.29
BV	63.8	26.7	19.7	60.1	10.4	0.074	0.034	19.6
Level I ^1^	90	40	35	100	15	0.20	0.15	35
Level II ^1^	250	60	100	300	25	0.60	1.0	350
Level III ^1^	350	200	400	500	30	1.0	1.5	500

^1^ Environmental quality standard for soil (GB 15618-1995).

**Table 3:** Comparison of PTEs (mg/kg) in roadside soils with the previous literature from China and other countries.

	Cr	Ni	Cu	Zn	As	Cd	Hg	Pb
Zhengzhou	65.58	24.27	22.88	88.57	10.97	0.41	0.08	24.76
Beijing, China [32]	60.3	23.3	31.3	83.8	8.55	0.174	0.316	33.7
Shanghai, China [10]	56.1	34.7	41.6	274.6	/	0.34	/	44.2
Xi’an, China [33]	75.1	22.7	27.4	67.7	10.9			23.9
Melbourne, Australia [34]	18–29	7–20	4–12	10.36–88.73	/	0.06–0.59	/	16–144
Siena, Italy [35]	70.9	40.3	52.3	142	/	0.31	/	104
Toronto, Canada [36]	36.0	/	21.0	/	/	0.54	/	17.0
Guwahati, India [37]	109.0	89.1	170.2	302.8	/	8.8	/	171.1

**Table 4:** Descriptive statistics for PTE concentrations (mg/kg) in river surface sediments samples of Zhengzhou.

	Cr	Ni	Cu	Zn	As	Cd	Hg	Pb
Max	93.63	51.78	75.31	281.00	15.28	1.66	0.22	71.49
Min	50.28	16.74	13.12	43.77	5.19	0.07	0.04	14.23
Mean	64.61	25.52	24.78	95.35	9.36	0.26	0.09	22.60
SD	9.44	5.77	13.49	55.26	2.34	0.25	0.05	9.97
CV	0.15	0.23	0.54	0.58	0.25	0.96	0.56	0.44
BV	63.80	26.70	19.7	60.10	10.40	0.074	0.034	19.60
TEL ^1^	43.4	22.7	31.6	121	9.8	0.99	0.18	35.8
PEL ^2^	111	48.6	149	459	33	5	1.1	128
ERL ^3^	81	20.9	34	150	8.2	1.2	0.15	46.7
ERM ^4^	370	51.6	270	410	70	9.6	0.71	218

^1^ Threshold effect level, ^2^ probable effect level, ^3^ effects range low, ^4^ effects range median.

**Table 5:** Mean concentration of PTEs (mg/kg) in urban river surface sediments in various sites of cities and rivers.

	Cr	Ni	Cu	Zn	As	Cd	Hg	Pb
Zhengzhou	64.61	25.52	24.78	95.35	9.36	0.26	0.09	22.60
Shaying River, China [43]	55.26	30.79	23.38	86.62	10.72	0.34	0.14	24.01
Shanghai, China [44]	106.3	/	87.5	296.8	/	0.53	0.42	35.2
Qingdao, China [45]	69.3	/	23.6	64.6	7.7	0.159	/	20.2
Wuhan, China [46]	134.2	48.3	/	357.4	16.6	1.1	0.39	58.0
Kaifeng, China [47]	67.86	28.46	290.65	1936.95	/	24.51	/	115.34
Lower Pearl River, USA [48]	19.1	/	14.0	49.0	2.3	0.0	/	29.6
Brisbane River, Australia [49]	15	15.3	29	106.6	3.6	0.3	0.4	25.6
Tsurumi River, Japan [50]	102.9	36.6	133.0	381.1	11.0	1.0	/	40.8

## Data Availability

Not applicable.

## Supplementary Materials

The following are available online at https://www.mdpi.com/article/10.3390/toxics11020140/s1, Figure S1: Study area and sampling sites in Zhengzhou, Table S1: The recovery rate of standard soil (North Plain soil (GSS-13)), Table S2: Contamination categories based on *I_geo_* values, Table S3: Grades of potential ecological risk.
