# Supplementary material for: A Comprehensive Exploration on Occurrence, Distribution and Risk Assessment of Potentially Toxic Elements in the Multi-Media Environment from Zhengzhou, China
Source: Toxics. 2023 Jan 31;11(2):140. doi: 10.3390/toxics11020140 (PMC9959330; doi:10.3390/toxics11020140)
Supplement: Supplementary file 1 [file toxics-11-00140-s001.zip › toxics-2110150-supplementary.pdf]

toxics

Supplementary Materials: A Comprehensive Exploration on Occurrence, Distribution and Risk Assessment of Potentially Toxic Elements in the Mul-ti-Media Environment from Zheng-zhou, China

Jialu Li, Qiting Zuo, Hao Hu, Feng Feng, Hongtao Jia and Yingxin Ji

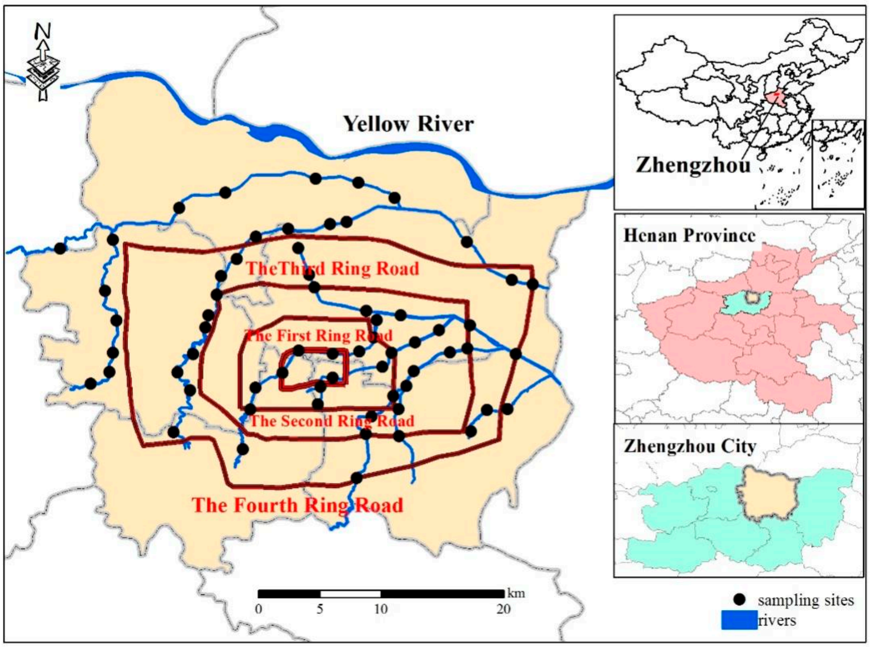

Figure S1. Study area and sampling sites in Zhengzhou.

Table S1. The recovery rate of standard soil (North Plain soil (GSS-13)).

	measured value (mg/kg)	standard value (mg/kg)	recovery rate (%)
Cr	64.08	65±2	98.59
Ni	27.47	28.5±1.2	96.40
Cu	20.57	21.6±0.8	95.22
Zn	70.18	65±3	107.97
As	10.92	10.6±0.8	103.04
Cd	0.14	0.13±0.01	109.84
Hg	0.49	0.052±0.006	93.97
Pb	22.52	21.6±1.2	104.24

Table S2. Contamination categories based on I_{geo} values.

I_{geo}	class	pollution level
≤ 0	0	Uncontaminated
0-1	1	Uncontaminated to moderately contaminated
1-2	2	Moderately contaminated
2-3	3	Moderately to heavily contaminated
3-4	4	Heavily contaminated

4-5	5	Heavily to extremely contaminated
> 5	6	Extremely contaminated
≤ 0	0	Uncontaminated

Table S3. Grades of potential ecological risk.

E_r^i		RI	
<40	low risk	<150	low risk
40-80	moderate risk	150-300	moderate risk
80-160	considerable risk	300-600	considerable risk
160-320	high risk	600-1200	high risk
>320	very high risk	>1200	very high risk
